# *Cis–Trans* Configuration of Coumaric Acid Acylation Affects the Spectral and Colorimetric Properties of Anthocyanins

**DOI:** 10.3390/molecules23030598

**Published:** 2018-03-07

**Authors:** Gregory T. Sigurdson, Peipei Tang, M. Mónica Giusti

**Affiliations:** Department of Food Science and Technology 2015 Fyffe Ct., The Ohio State University, Columbus, OH 43210-1007, USA; sigurdson.5@osu.edu (G.T.S.); tang.451@osu.edu (P.T.)

**Keywords:** delphinidin, petunidin, natural color, isomers, *cis* acylation, *trans* acylation, *Solanum melongena* L., *Lycium ruthenicum*

## Abstract

The color expression of anthocyanins can be affected by a variety of environmental factors and structural characteristics. Anthocyanin acylation (type and number of acids) is known to be key, but the influence of acyl isomers (with unique stereochemistries) remains to be explored. The objective of this study was to investigate the effects of *cis–trans* configuration of the acylating group on the spectral and colorimetric properties of anthocyanins. Petunidin-3-rutinoside-5-glucoside (Pt-3-rut-5-glu) and Delphinidin-3-rutinoside-5-glucoside (Dp-3-rut-5-glu) and their *cis* and *trans* coumaroylated derivatives were isolated from black goji and eggplant, diluted in pH 1–9 buffers, and analyzed spectrophotometrically (380–700 nm) and colorimetrically (CIELAB) during 72 h of storage (25 °C, dark). The stereochemistry of the acylating group strongly impacted the spectra, color, and stability of the Dp and Pt anthocyanins. *Cis* acylated pigments exhibited the greatest λ_max_ in all pH, as much as 66 nm greater than their *trans* counterparts, showing bluer hues. *Cis* acylation seemed to reduce hydration across pH, increasing color intensity, while *trans* acylation generally improved color retention over time. Dp-3-*cis-p*-cou-rut-5-glu exhibited blue hues even in pH 5 (C*_ab_ = 10, h_ab_ = 256°) where anthocyanins are typically colorless. *Cis* or *trans* double bond configurations of the acylating group affected anthocyanin spectral and stability properties.

## 1. Introduction

The color of fresh fruits and vegetables and their food products relates to their overall market sales and success. Consumers can infer a variety of attributes such as flavor, safety, nutritional value, and more due to the color of a product. Due to links between certain synthetic colorants and hyperactivity in children, allergenicity in sensitive populations, and demand for more natural products, the use of naturally derived colorants in foods has been increasing [1,2]. Colorants derived from nature now lead in usage in foods having demonstrated a 77% growth from 2009 to 2013 [3]. Colorants can be derived from many natural sources including plants, microorganisms, animals, and also minerals [4]. Of plant-derived pigments, anthocyanins comprise the largest group of water-soluble pigments; more than 700 structures have been identified [5]. They provide unique advantages and challenges to food producers as they are responsible for a wide range of colors in nature including orange, red, purple, and blue. Individual anthocyanins are also widely known to express different hues based upon structural changes that depend on environmental pH and chemical substitution patterns. 

Several structural factors including chromophore methoxylation, hydroxylation, glycosylation, and acylation all affect the colors expressed by anthocyanins. The six most common anthocyanidins in edible produce differ in degree of B-ring hydroxylation and/or methoxylation, their hues typically being bluer with increasing degree of substitution [6]. Methoxylation of the chromophore, compared with hydroxylation, results in slightly redder hues; the h_ab_ values were demonstrated to be negatively correlated with not only the total anthocyanin content of processed blueberry juice but also the delphinidin/malvidin ratio [7]. Glycosylation of cyanidin at C3 typically intensified and stabilized the color of anthocyanidins in most pH; however, glycosylation of cyanidin at C3 and C5 has been reported to decrease pK_h_ and color stability [8,9,10,11]. Despite the decreased color stability, this glycosylation pattern also resulted in hue shifts toward purple-blue tones [12,13]. 

Acylation has been considered an important aspect regarding the color expression and stability of many anthocyanins [14]. The chemical mechanisms affecting colorimetric properties are believed to be complex and may include intramolecular copigmentation, steric hindrance protecting the chromophore from hydration, extension of the electron delocalization system, and further alterations in the geometric properties of the molecule [13,15,16]. Attachment of hydroxycinnamic acids typically results in an increase in pK_h_ and, therefore, increased color expression in comparatively higher pH, which contributes to the predominance of acylated anthocyanin colorants in the food industry [13,14]. Acylation of cyanidin derivatives with cinnamic acids has been reported to induce a hue shift towards purple. Even the location of the acyl attachment plays an important role in colorimetric properties; λ_max_ differences as large as 57 nm were found in derivatives of cyanindin-3-sinapoyl-sophoroside-5-glucoside due to attachment of the sinapoyl moiety on different locations of the sophorosoyl moiety [17]. Aromatically acylated anthocyanins are typically found esterified to *trans*-configured variants of hydroxycinnamic acids; however, few reports demonstrate the existence of *cis-*configured hydroxycinnamic acylated anthocyanins. 

When found in nature, the *cis* and *trans* acylated forms are found together, but the *trans* acylated anthocyanin always predominates [18]. Only 12 naturally occurring *cis* acylated anthocyanin derivatives were identified in reviewing the current literature; however, photoirradiation was demonstrated to induce *trans* to *cis* isomerization in vitro [18,19,20,21,22,23]. Naturally occurring *cis* acylated anthocyanins are primarily reported in parts of plants that receive greater amounts of light, such as flowers, leaves, and some fruits [18,19,20,21,22,23]. Of botanical and edible fruits, the black goji (*Lycium ruthenicum*), Asian eggplant (*Solanum melongena* L.), and purple bell peppers (*Capsicum annuum* L.) have been previous identified as natural sources of *cis* coumaric acylated petunidin and delphinidin [21,24]. Therefore, these materials may serve as sources of naturally derived food colorants; however, few reports compare the spectral properties of the isolated pigments. Of those, they were characterized primarily in acidic methanol or aqueous solutions at acidic pH (≤4.6) [18,19,20].

Structural modifications of the same chromophore result in unique colorimetric properties of each pigment, which complicates selection of pigment sources or specific pigments to obtain desired colors in food products. Therefore, there is a need to better understand how these structural components impact the color of these pigments in a wide range of conditions. Very few reports were found that compared spectral and colorimetric properties of *cis* and *trans* isomers of hydroxycinnamic acyl moieties on the same anthocyanin in acidic pH and none in alkaline pH. Therefore, the objective of this work was to investigate the impact of isomeric configurations of acyl moieties on the color expression of delphinidin and petunidin anthocyanins. 

## 2. Results and Discussion

**Figure 1:**
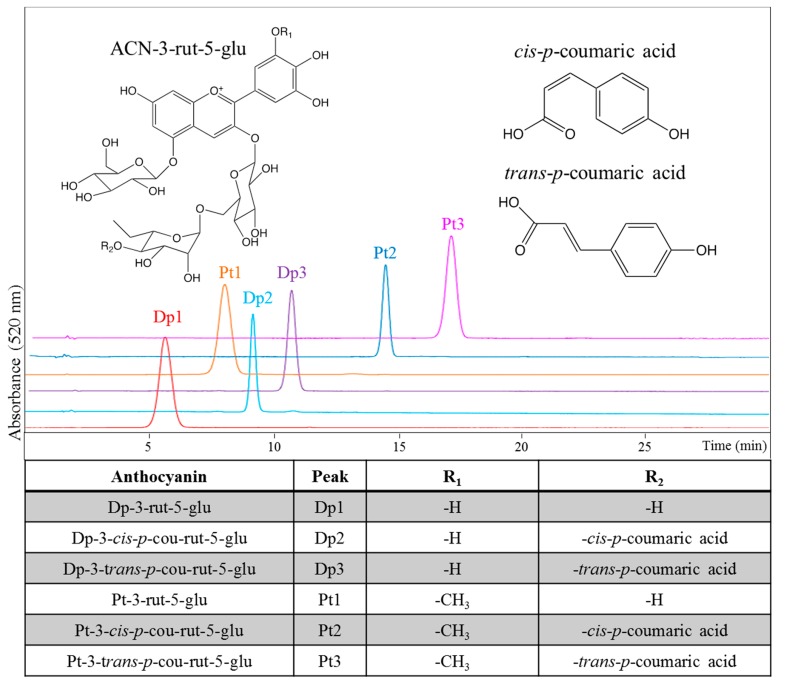
HPLC chromatograms (detection at 520 nm), identities, and structures of isolated delphinidin (Dp) and petunidin (Pt) derivatives.

Despite the relative rarity of *cis* acylated anthocyanins in nature, two food sources of *cis* and *trans* acylated anthocyanins were identified for use in this study [21,22,24]. The *cis* and *trans p*-coumaroylated isomers of delphinidin-3-rutinoside-5-glucoside (Dp-3-rut-5-glu) were predominant in Asian eggplant (*Solanum melongena* L.) extracts, while the *cis* and *trans p*-coumaroylated derivatives of petunidin-3-rutinoside-5-glucoside (Pt-3-rut-5-glu) were found in black goji (*Lycium ruthenicum*). Alkaline saponification of the extracts yielded predominantly Dp-3-rut-5-glu and Pt-3-rut-5-glu, as observed in HPLC chromatograms and identified in Figure 1.

### 2.1. Spectrophotometric Properties of Acylated Anthocyanin Derivatives

**Figure 2:**
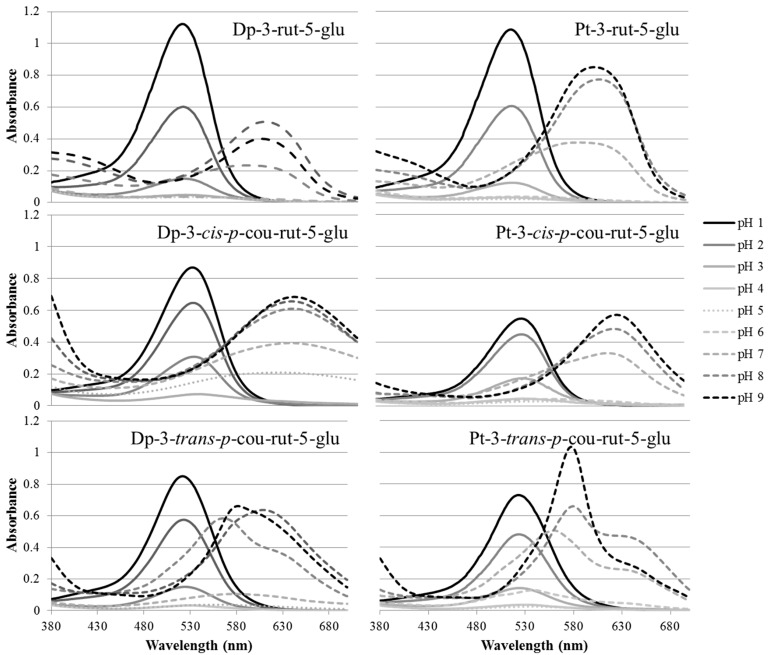
Visible absorbance (380–700 nm) of isolated delphinidin (Dp) and petunidin (Pt) derivatives, pH 1–9.

The differences in the acyl substitutions of these anthocyanins resulted in unique differences in visible light absorption spectra that became more pronounced as pH was increased. In very acidic pH, the spectral absorbance curves in a family of pigments were fairly similar, exhibiting similar low absorbance approaching the UV region and having little to no absorbance approaching the IR region (Figure 2). Anthocyanins glycosylated at only C3 are well documented to exhibit characteristic high proportional absorbance between 420 and 440 nm compared with the absorbance at the λ_max_ in acidic pH; however, diglycosylation at C3 and C5 results in a decrease in this absorbance between 420 and 450 nm when compared with anthocyanins glycosylated at only C3 [25]. The absorbance spectra of these Dp and Pt derivatives of this study, even the acylated ones, are consistent with these reported trends (Figure 2).

**Figure 3:**
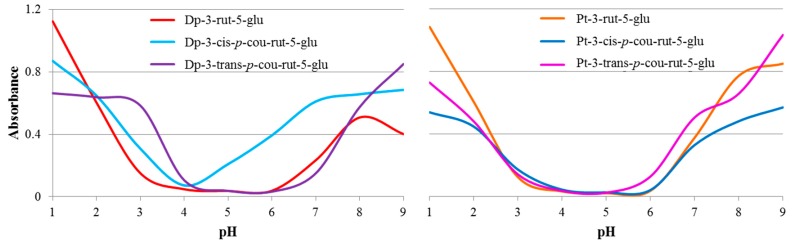
Absorbance of delphinidin (Dp) and petunidin (Pt) derivatives at respective λ_max_ in pH 1–9.

Typical of anthocyanins, the formation of predominantly colorless anthocyanin derivatives occurred in mildly acidic pH (4–6) for all derivatives (Figure 2 and Figure 3) resulting in low absorbance and almost linear spectra. Nonacylated anthocyanin derivatives were expected to be most prone to hydration and formation of colorless structures. Dp-3-rut-5-glu showed the lowest color retention in the most pH values (Table 1); however, the *trans* acylated counterpart showed similar color bleaching. The *cis* acylated Dp isomer generally showed significantly higher color expression than the others in the series (Table 1 and Figure 3). In the case of Mv-3-*p*-cou-rut-5-malonyl-5-glu, hydroxycinnamic acyl moieties in the *cis* conformation are believed to be stereochemically nearly parallel to the anthocyanidin chromophore; while in *trans* configuration, the acyl moiety lies quasi-perpendicular to the aglycones [18]. This difference in stereochemistry may play important roles in the protection of the chromophore against hydration and, therefore, color loss. This trend was not consistent for the Pt derivatives. The *cis* acylated isomer showed slightly more color loss than did the *trans* acylated isomer in pH 6–9, but both bleached less than the nonacylated counterpart (Table 1). Perhaps the slightly bulkier methoxyl attachment on the B-ring of Pt played a role in protection from hydration.

As pH increased to ≥6, the λ_max_ of the pigments was bathochromically shifted, and the spectral characteristics between the different species became more pronounced. Anthocyanins exist in a structurally dynamic equilibrium that is pH dependent; while in acidic pH, they exist primarily in cationic flavylium or hemiketal forms that appear red or colorless. With increasing pH to neutral and alkaline pH, quinoidal structures begin to predominate, which can become anionic, but also have greater λ_max_ and express purple and blue hues [26]. Interestingly, the absorbance spectra between the *cis* and *trans* acylated isomers of Dp-3-rut-5-glu and of Pt-3-rut-5-glu exhibited many differences (Figure 2). The *cis* acylated isomers exhibited a major peak in either acidic or alkaline pH. The peak of Dp-3-*cis*-*p*-cou-rut-5-glu was fairly broad in alkaline pH and was comparably sharper for Pt-3-*cis*-*p*-cou-rut-5-glu. In pH 7–9, the *trans* acylated isomers exhibited prominent absorbance shoulders in greater wavelengths than the λ_max_. Interestingly, these absorbance spectra resembled mirror images of the spectra of anthocyanin-3-glucosides in acidic pH, in which they exhibit a characteristic absorbance shoulder at 420–450 nm [25]. The differences in stereochemistry between the *cis* and *trans* acylated isomers likely played a role in the unique absorbance, perhaps through intramolecular copigmentation or by structural distortions of the aglycone (stretching, bending, or torsion) that modify the π-delocalization of the chromophore by molecular substitution [16].

The λ_max_ of the different acylated derivatives also varied considerably not only across pH but also when comparing one to another in the same pH (Table 1). Generally, the λ_max_ of acylated derivatives were greater than those of the nonacylated derivatives. The impact of the different stereochemistry between the *cis* and *trans* configuration of coumaric acid on the λ_max_ of Dp and Pt derivatives was pronounced. In all pH, the λ_max_ of Dp-3-*cis*-*p*-cou-rut-5-glu was greater than the *trans* acylated counterpart as well as all other Dp derivatives (Table 1). This was also the case with acylated Pt except in pH 4; at which the pigments were almost completely bleached in this pH. This was consistent with previous comparisons of *cis* and *trans* isomer acylated anthocyanins in acidic pH or acidified methanol [18,19,20]. In pH 5, the λ_max_ and absorbance of Dp-3-*cis*-*p*-cou-rut-5-glu was surprisingly large (618 nm, Figure 3), 36 nm greater than Dp-3-*trans*-*p*-cou-rut-5-glu in the same pH. This is an atypically large λ_max_ for anthocyanins in acidic pH, especially without the presence of other cofactors such as metal ions [27]. Pt-3-*cis*-*p*-cou-rut-5-glu also showed rather large λ_max_ of 621–630 nm in neutral and alkaline conditions, 48–62 nm greater than the large λ_max_ of the *trans* acylated counterpart. In this pH range (6–9), Dp-3-*cis*-*p*-cou-rut-5-glu exhibited λ_max_ 23–66 nm greater than its *trans* acylated counterpart.

### 2.2. Colorimetric Properties of Acylated Anthocyanin Derivatives

**Figure 4:**
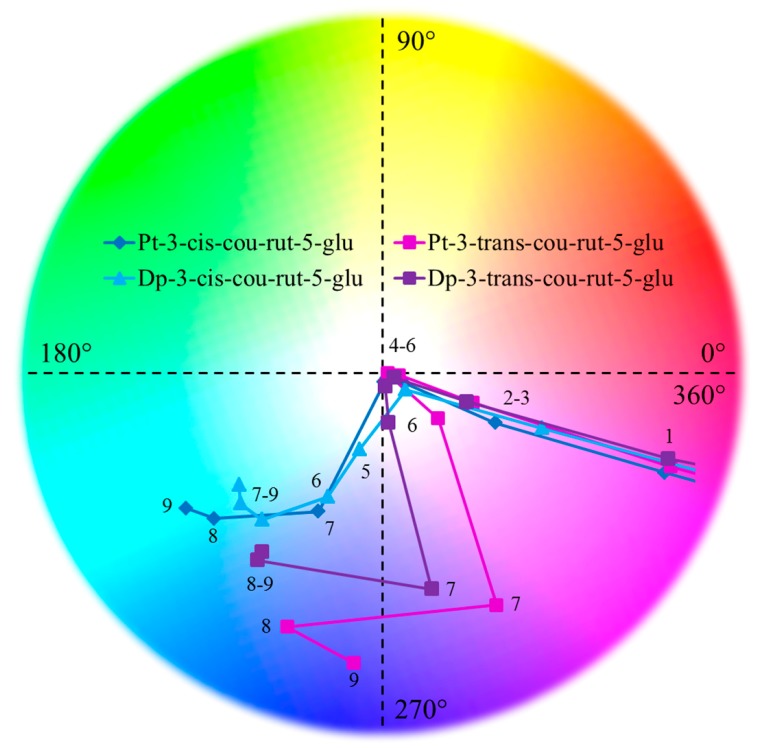
Hue angle (arctan b*/a*) of *cis* and *trans* acylated delphinidin (Dp) and petunidin (Pt) derivatives, pH 1–9.

The diversity in absorbance spectra of these different anthocyanins resulted in unique hues being expressed by of each of the pigments (Table 2 and Figure 4). Due to lack of molar absorptivity coefficients for each of these individual pigments, they were quantified as equivalents of Cy-3-diglucoside-5-glucoside or coumaroylated derivatives, as described in Section 3.2.4. Therefore, differences in the colorimetric data, primarily L* (luminosity/lightness) and C*_ab_ (chroma/intensity), could be affected by the differences in concentration. L* and C*_ab_ values were comparable between the different derivatives in the same pH and also between the derivatives of the two anthocyanidin series, displaying similar respective trends over the pH conditions tested. However, in alkaline pH, Pt derivatives showed slightly smaller L* and larger C*_ab_ values, and slightly darker and more intense colors (Table 2). Depending on pH, differences in L* values ranged 1.2–14.4 units and ranged 2.0–19.2 for C*_ab_ values for Dp derivatives. For Pt derivatives, differences in L* ranged 0.3–13.4 and 0.5–14.1 for C*_ab_. Differences were typically largest in neutral and alkaline pH and were less pronounced in acidic pH. However, in acidic pH, L* values ranged less than 10 units between different pigments in the same pH. Ranges in C*_ab_ were greater, demonstrating the pigments showing different color intensities. In order to account for these variances, data were normalized by calculating % absorbance retention, defined as Absorbance at respective λ_max_ in pH_n_ / Absorbance in pH_1_ × 100 (Table 1). Acylation typically worked to decrease L* and increase C*_ab_ values, darkening and intensifying color. The effects of the acyl isomerization showed opposite effects in acidic and alkaline pH. In acidic pH, the *cis* acylated isomers generally showed lower L* values and greater C*_ab_ values, but in alkaline pH, the *trans* acylated isomers typically expressed these traits.

The hues of these derivatives varied more greatly as a function of the isomeric acylation patterns than of the type of aglycone. As expected of anthocyanins, reddish hues were expressed by these anthocyanins in acidic pH. Acylation typically altered the hue to be comparatively pinker in acidic pH, leading to hues ~340° for all acylated derivatives in pH 1–3 (Table 2). As pH was increased from 1 to 6, all the Dp and Pt derivatives became extremely faint (almost colorless), which led to some variability in hue. The hues of the nonacylated derivatives fell in the red-yellow region (26.3–75.7°) in pH 4–6 while the acylated derivatives were varied more considerably from pink to purple to blue. In pH ≥ 7, these Dp derivatives expressed blue colorations (hue 180–270°) while the Pt derivatives were blue and purple (hue 215–297°), suggesting that the methoxyl group on the B-ring of Pt increased the redness of the chromophore. Although the hues of the acylated isomers of the same chromophore were similar in acidic pH 1–3, they differed significantly in pH ≥ 4. Dp-3-*cis*-*p*-cou-rut-5-glu expressed blue colorations in the widest pH range of 5–9. Blue colorations expressed by anthocyanins in such acidic pH are uncommon and often require additional co-factors such as metal ions or polyacylation [28,29]. Dp-3-*trans*-*p*-cou-rut-5-glu, despite bearing the same acyl moiety, expressed comparatively more purple colorations (Figure 4 and Table 2). Similar observations were observed between the *cis* and *trans* acylated Pt derivatives. Hues of the *trans* acylated derivatives expressed more purple hues (hues = 250–297°) while Pt-3-*cis*-*p*-cou-rut-5-glu expressed blues in pH ≥ 7 (hues = 215–256°) (Table 2). The *cis–trans* isomerization of acyl moieties on anthocyanins played significant roles in the color expression of the same anthocyanin, affecting both hydration (colorlessness) and hue.

### 2.3. Color Stability over Time

Stability of anthocyanins is inherently related to the structures of the pigments due to chemical substitution patterns of the chromophore and also the dynamic structural equilibria in which they exist as a function of pH. Table 3 displays the half-life calculations based on change in absorbance (as measured at the λ_max_ from t_0_) during dark, ambient storage. As would be expected, stability of the Dp and Pt derivatives was decreased as pH was increased—in some cases, by almost a 950-fold decrease from pH 1 to pH 9 (Table 3). Dp as aglycone is known to be reactive due to the presence of reactive hydroxyl groups on the B-ring; interestingly, Dp-3-rut-5-glu and its derivatives generally exhibited greater half-lives than did Pt-3-rut-5-glu derivatives in acidic conditions.

Overall, acylation demonstrated stabilizing effects on Dp-3-rut-5-glu and Pt-3-rut-5-glu, increasing half-lives in most pH levels evaluated. Increases in storage stability by acylation were most pronounced in alkaline pH, leading to 19.6× and 5× increases in half-life in pH 9 when comparing Dp-3-*trans*-*p*-cou-rut-5-glu to Dp-3-rut-5-glu and Pt-3-*trans*-*p*-cou-rut-5-glu to Pt-3-rut-5-glu, respectively. The *cis* or *trans* conformation of the acyl moiety also uniquely impacted the stability for the acylated Dp-3-rut-5-glu and Pt-3-rut-5-glu derivatives. In very acidic pH 1, the half-life of Dp-3-*cis*-*p*-cou-rut-5-glu was greater than that of its *trans* counterpart while the half-life of Pt-3-*trans*-*p*-cou-rut-5-glu was greater than that of its *cis* form. However, in pH > 1, half-lives of both Dp-3-*trans*-*p*-cou-rut-5-glu and Pt-3-*trans*-*p*-cou-rut-5-glu were greater than those of the *cis* acylated counterparts. While the *cis* acylated isomers of Dp and Mv have increased color stability across pH related to protection of the chromophore against hydration [18], the retention of color over time was found to be decreased when comparing *cis* to *trans* acylated isomers. These would be important considerations in the selection of specific anthocyanins depending on the application. In low-acid (pH 4–6) products in which the pigments will be dried, such as candy panning or extruded ready-to-eat cereals, *cis* acylated anthocyanins may provide more intense coloration. However, in high moisture and neutral products such as protein or dairy beverages, anthocyanins are more labile to degradation during storage; thus, *trans* acylated anthocyanins may express colorations for longer amounts of time. 

Interestingly, all the Pt derivatives (acylated or not) showed greater stability in pH 8 than in neutral pH 7 or in alkaline pH 9, which might be explained by anthocyanins’ structural transformation according to the pH environment. In mildly acidic and neutral pH, the flavylium cation can be hydrated (colorless) or deprotonated and exist as different quinonoidal base forms. With subsequent increases in pH, the quinonoidal base can be ionized to have one or two negative charges [30]. The kinetic parameters pK_a2_ and pK_a3_ are used to denote the dissociation constants for the transformations from the neutral quinonoidal base to having one and two negative charges, respectively. The pK_a2_ and pK_a3_ of the Pt aglycone were found to be pH 6.99 and 8.27, respectively [31]. Thus, a large proportion of blue quinonoidal base forms with one negative charge exist, which may be more prone to degradation at pH 7, while at pH 8, a higher proportion of quinonoidal bases with 2 charges exists, further modifying the reactivity of the pigment. Nevertheless, the retention of color over time was found to be enhanced by acylation, and its orientation in space proved another important consideration. 

## 3. Materials and Methods 

### 3.1. Materials 

Delphinidin- and petunidin-rich anthocyanin extracts were prepared from the peels of Asian eggplant varieties (*Solanum melongena* L.) and dried black goji fruits (*Lycium ruthenicum*) purchased from grocery stores (respectively, Columbus, OH, USA and Shanghai, China). 

Tris(hydroxymethyl)aminomethane, 99%, was purchased from Alfa Aesar (Ward Hill, MA, USA). All other chemicals and solvents were ACS or HPLC grade and purchased from Fisher Scientific (Fair Lawn, NJ, USA). 

### 3.2. Methods

#### 3.2.1. Anthocyanin Preparation for Pigment Isolation

Anthocyanins were extracted from the skins of eggplants and black goji fruits with acidified 70% acetone (1.5% trifluoroacetic acid and 0.01% HCl, respectively), isolated by phase partition with water and chloroform, and purified by C_18_ solid-phase extraction with acidified water and ethyl acetate following procedures described by Rodríguez-Saona and Wrolstad [32]. 

To obtain nonacylated counterparts, aliquots from each extract were dissolved in 10 mL of 10% KOH for 10 min to cleave the ester bonds between the acyl and glycosyl moieties of the anthocyanins, according to procedures of Giusti and Wrolstad [33]. The extracts were again purified and concentrated by solid-phase extraction described above.

#### 3.2.2. Anthocyanin Isolation 

Dp-3-rut-5-glu and the *cis* and *trans* acyl isomers of Dp-3-*p*-cou-rut-5-glu were isolated from Asian eggplant anthocyanin extracts while Pt-3-rut-5-glu, Pt-3-*cis*-*p*-cou-rut-5-glu, and Pt-3-*trans*-*p*-cou-rut-5-glu were isolated from the black goji extracts by semipreparative reverse-phase HPLC. The system was produced by Shimadzu (Columbia, MD, USA) and composed of LC-6AD pumps, a CBM-20A communication module, an SIL-20A HT autosampler, a CTO-20A column oven, and an SPD-M20A Photodiode Array detector. LCMS Solution Software (Version 3, Shimadzu, Columbia, MD, USA) was used to monitor samples. 

Anthocyanins were separated by a Luna reverse-phase pentafluorophenyl (PFP2) column with 5 µm particle size and 100 Å pore size in a 250 × 21.2 mm column (Phenomenex^®^, Torrance, CA, USA). The flow rate was 10.0 mL/min for a run time of 30 min. A binary gradient was used with solvents A: 4.5% formic acid in water and B: acetonitrile. For nonacylated Dp, the gradient began at 8% B and was constant for 1 min, then increased to 16.5% B over 31 min, while for acylated Dp, the gradient began at 10% B and increased to 30% by 30 min. Similarly, the gradient for nonacylated Pt began at 10% B and was constant for 1 min then increased to 15% B over 31 min. Acylated Pt was separated with a more complex gradient starting at constant 12% B for 2 min, then increasing to 21% B over 25 min and being held constant until 30 min, before finally increasing to 30% B by 50 min. Elution of anthocyanins was monitored at 520 nm, and desired peaks were collected manually. Isolates were diluted with 2–3 volumes of water and subjected to solid phase extraction to remove excess formic acid and concentrate the pigments.

#### 3.2.3. Isolated Anthocyanin Identity—HPLC-MS

The identity of the pigments was monitored by analytical reverse-phase HPLC-MS with a similar system (Shimadzu, Columbia, MD, USA) differing by pumps LC-20AD and including an LCMS-2010 mass spectrometer. The analytical column was a Kinetix reverse-phase pentafluorophenyl (PFP2) column with 2.6 µm particle size and 100 Å pore size in a 100 × 4.6 mm column (Phenomenex®, Torrance, CA, USA). The flow rate was 0.6 mL/min for a binary gradient consisting of solvents A: 4.5% formic acid in water and B: acetonitrile. The gradient for all Dp derivatives began at 0% B, increased 0–10% from 0 to 1 min and then 10–23% B from 1 to 30 min; for Pt derivatives, the gradient began at 7% B for 2 min and up to 20% B over 30 min. Spectral data was collected at 200–700 nm. A quantity of 0.13 mL per minute was diverted to the mass spectrometer. Mass spectrometry was conducted under the positive ion mode; data were collected from *m*/*z* 200–1200. Mass spectrometry, order of elution, and comparison to literature were used to identify the anthocyanins [22,29,34,35]. The structures of the predominant and isolated anthocyanins of the extracts may be found in Figure 1.

#### 3.2.4. Sample Preparation 

The anthocyanin extracts were diluted to concentrations of 50 µM in buffers of pH 1–9 ± 0.05. Buffer systems were composed of 0.025 M KCl for pH 1–2, 0.1 M sodium acetate for pH 3–6, 0.25 M TRIS for pH 7–8, and 0.1 M sodium bicarbonate for pH 9. The pH of the buffer systems was adjusted with concentrated HCl or 10% NaOH prior to final volume adjustment. Anthocyanin concentrations of the extracts and isolates were determined by the pH differential methodology [36]. Dp-3-rut-5-glu and Pt-3-rut-5-glu isolates were expressed as cyanidin-3-diglucoside-5-glucoside equivalents, while the Dp-3-*p*-cou-rut-5-glu and Pt-3-*p*-cou-rut-5-glu isolates were expressed as Cy-3-*p-*cou-diglu-5-glu equivalents using ε reported by Ahmadiani et al [17]. After anthocyanins were diluted in buffers, the pH of every sample was verified to be ± 0.05 using a Mettler Toledo Inc. S220 SevenCompact™ pH/Ion meter (Columbus, OH, USA). Samples were equilibrated at room temperature in the dark for 30 min prior to initial analysis. Samples were then sealed and stored in the dark for 72 h at 25 °C to briefly assess the color stability of the pigments. Three replicates were prepared for each treatment. 

#### 3.2.5. Visible Spectrophotometry of Samples 

After pigment dilution, samples were equilibrated at room temperature in the dark for 30 min prior to initial spectrophotometric analysis, referred to as t_0_. Aliquots of 300 µL of each sample were transferred to poly-d-lysine-coated polystyrene 96-well plates and evaluated by visible absorbance (380–700 nm, 1 nm intervals) spectrophotometry with a SpectraMax 190 Microplate Reader (Molecular Devices, Sunnyvale, CA, USA). Color intensity of the pigments in pH 1–9 was also compared by % absorbance retention calculated as Absorbance in pH_n_/Absorbance in pH_1_ at respective λ_max_ × 100. Most anthocyanins express a high color intensity in very acidic pH ≤ 1; therefore, this proportion was used as a measure to compare the degree of color loss in different pH.

#### 3.2.6. Colorimetry of Samples

Colorimetric data was expressed in the CIELAB communication system, the official color scale from the CIE (Commission internationale de l’éclairage), where LAB stands the color coordinates in the color space. Color parameters were calculated from spectral absorbance data (380–700 nm, 5 nm intervals) using the ColorBySpectra software [37] based on standard 1964 CIE equations, D_65_ illuminant spectral distribution, and 10° observer angle functions. Color values are expressed as a function of L* (luminosity/lightness), C*_ab_ (chroma/intensity) and h*_ab_ (hue angle).

#### 3.2.7. Calculation of Sample Half-Lives

Samples in the well plates were kept for 72 h at room temperature and in the dark to compare the relative stabilities of the different spatial configurations of the acylated anthocyanins. Plates were closed with a lid and sealed with parafilm to prevent evaporation during storage. Absorbance and color measurements taken after 30 min equilibration were used as time 0, t_0_. Subsequent readings were taken throughout the storage at times 2, 4, 6, 24, 48, and 72 h . Linear regressions were prepared from the natural logarithm of the absorbance (at the λ_max_ from t_0_) during the time points of the study, following the formula ln[A_t_] = –kt + ln[A_t0_]. Linear regressions showed coefficient of determination values (R^2^ values ≥ 0.89). The half-life (t_1/2_) was calculated for each of the samples as t_1/2_ = ln 2/k.

#### 3.2.8. Statistical Evaluation of Data 

Figures, means, and standard deviations were produced using Microsoft Office Excel 2010 (Office 14.0, Microsoft. Redmond, WA, USA). The λ_max_, % absorbance retention, and half-lives of the different pigments were evaluated by one-way analysis of variance (ANOVA) (two-tailed, α = 0.05) and post hoc Tukey’s test (α = 0.05) using Minitab 16 (Minitab Inc., State College, PA, USA). Of colorimetric data, only hue angles* were statistically compared by one-way analysis of variance (ANOVA) (two-tailed, α = 0.05) and post hoc Tukey’s test (α = 0.05). Due to differences in exact pigments’ concentrations, differences in L* and C*_ab_ values were expected and could not be equally compared statistically; the hue angle* was considered to be less variable due to small differences in concentration.

## 4. Conclusions

The stereochemistry of coumaric acid acylation had a strong impact on the spectra, color, and stability of Dp and Pt anthocyanins. Pigments with *cis* isomeric acylation had the greatest λ_max_ in all pH—as much as 66 nm greater than *trans* counterparts. Therefore, *cis* acylated isomers expressed bluer hues while the *trans* isomers were comparatively redder. Acylation with *trans* isomers induced a spectral shoulder in wavelengths ~630 nm off of the peak at the λ_max_ in pH ≥ 7, while *cis* isomers were documented by a broad main peak around these wavelengths. *Cis* acylation seemed to better protect the molecule against hydration, resulting in higher color expression across pH while *trans* acylation generally improved color retention over time. Anthocyanins typically express low color intensity in the mildly acidic pH common to many foods and confectionaries; *cis* acylated anthocyanins may help to fill this industry gap. In addition, it was found that Dp-3-*cis*-*p*-cou-rut-5-glu expressed the greatest λ_max_ (617–632 nm) in the widest pH range (5–9) compared with Pt-3-*cis*-*p*-cou-rut-5-glu with λ_max_ 553–630 nm in pH 5–9. Dp-3-*cis-p*-cou-rut-5-glu exhibited blue hues even in pH 5 (C*_ab_ = 10, h_ab_ = 256°) in which most anthocyanins exist in colorless hemiketal forms. Color stability across pH and over time of all derivatives was negatively impacted with increased pH; however, acylation improved stability in both of these aspects. This study has demonstrated the unique spectral properties and variety of hues of anthocyanins composed of the same chemical substitutions as to be a result of differing spatial configurations of the acyl moieties. It provides additional insight on the chemical attributes that affect the large diversity of hues expressed by anthocyanins in natural plant systems.

## Figures and Tables

**Table 1:** λ_max_ (nm) and % absorbance retention, defined as absorbance at respective λ_max_ in pH_n_/absorbance in pH_1_ × 100, of delphinidin (Dp) and petunidin (Pt) derivatives in pH 1–9, *n* = 3 (standard deviation in parenthesis). Different superscript letters indicate significant differences (*p* < 0.05) between derivatives of the same anthocyanidin in the same pH.

Anthocyanin	pH 1	pH 2	pH 3	pH 4	pH 5	pH 6	pH 7	pH 8	pH 9
**λ_max_** **(nm)**									
**Dp-3-rut-5-glu**	517 ^c^ (0)	518 ^c^ (0)	518 ^c^ (2)	521 ^c^ (0)	522 ^c^ (4)	450 ^c^ (1)	583 ^b^ (0)	603 ^c^ (1)	600 ^b^ (1)
**Dp-3-*cis*-*p*** **-cou-rut-5-glu**	528 ^a^ (1)	528 ^a^ (1)	529 ^a^ (1)	534 ^a^ (2)	617 ^a^ (1)	630 ^a^ (1)	632 ^a^ (0)	631 ^a^ (1)	632 ^a^ (1)
**Dp-3-*trans*-*p­*cou-rut-5-glu**	521 ^b^ (1)	523 ^b^ (0)	523 ^b^ (1)	531 ^b^ (1)	563 ^b^ (2)	576 ^b^ (1)	566 ^c^ (0)	608 ^b^ (2)	584 ^c^ (4)
**Pt-3-rut-5-glu**	518 ^c^ (0)	519 ^c^ (0)	521 ^c^ (2)	519 ^b^ (6)	522 ^b^ (9)	524 ^c^ (2)	589 ^b^ (1)	610 ^b^ (1)	604 ^b^ (1)
**Pt-3-*cis*-*p***-**cou-rut-5-glu**	531 ^a^ (0)	531 ^a^ (0)	532 ^a^ (1)	534 ^a^ (0)	553 ^a^ (6)	564 ^a^ (1)	621 ^a^ (1)	627 ^a^ (0)	630 ^a^ (1)
**Pt-3-*trans*-*p-*cou-rut-5-glu**	523 ^b^ (0)	524 ^b^ (1)	525 ^b^ (2)	535 ^a^ (4)	535^b^ (2)	538 ^b^ (0)	559 ^c^ (0)	579 ^c^ (0)	578 ^c^ (0)
**% absorbance retention**									
**Dp-3-rut-5-glu**		53.7 ^c^ (2.4)	13.4 ^c^ (0.1)	4.3 ^b^ (0.1)	3.3 ^c^ (0.4)	3.4 ^c^ (0.0)	20.8 ^b^ (0.3)	45.2 ^b^ (1.6)	35.7 ^b^ (4.2)
**Dp-3-*cis*-*p*-cou-rut-5-glu**		77.0 ^a^ (1.1)	36.3 ^a^ (1.2)	9.0 ^a^ (0.3)	25.6 ^a^ (0.8)	45.8 ^a^ (2.5)	70.6 ^a^ (2.2)	70.6 ^a^ (2.3)	71.1 ^a^ (1.3)
**Dp-3-*trans*-*p­*cou-rut-5-glu**		68.2 ^b^ (0.7)	18.6 ^b^ (0.2)	4.1 ^b^ (0.1)	4.9 ^b^ (0.2)	14.3 ^b^ (0.2)	70.5 ^a^ (2.4)	73.5 ^a^ (0.2)	68.4 ^a^ (3.0)
**Pt-3-rut-5-glu**		55.9 ^c^ (2.1)	11.5 ^c^ (0.2)	3.1 ^c^ (0.2)	2.1 ^c^ (0.1)	3.5 ^c^ (0.1)	34.8 ^c^ (4.1)	71.3 ^b^ (4.7)	78.3 ^c^ (3.1)
**Pt-3-*cis*-*p*-cou-rut-5-glu**		82.0 ^a^ (1.8)	31.8 ^a^ (0.4)	8.3 ^a^ (0.3)	5.1 ^a^ (0.1)	7.9 ^b^ (1.0)	60.4 ^b^ (3.3)	88.3 ^a^ (0.6)	104.4 ^b^ (0.7)
**Pt-3-*trans*-*p*-cou-rut-5-glu**		66.1 ^b^ (0.9)	19.6 ^b^ (1.1)	5.1 ^b^ (0.3)	3.5 ^b^ (0.3)	17.8 ^a^ (0.7)	69.4 ^a^ (0.8)	90.3 ^a^ (3.4)	141.8 ^a^ (4.8)

**Table 2:** CIE-L*C*_ab_h_ab_ colorimetric values (standard deviation) of delphinidin (Dp) and petunidin (Pt) derivatives in pH 1–9, *n* = 3. Different superscript letters indicate significant differences (*p* < 0.05) between derivatives of the same anthocyanidin in the same pH.

Anthocyanin	pH 1	pH 2	pH 3	pH 4	pH 5	pH 6	pH 7	pH 8	pH 9
**Lightness** (L*)									
**Dp-3-rut-5-glu**	74.1 (0.1)	82.2 (0.5)	93.7 (0.1)	97.2 (0.1)	97.5 (0.2)	97.2 (0.0)	84.4 (0.3)	77.3 (0.4)	80.5 (1.5)
**Dp-3-*cis*-*p­*cou-rut-5-glu**	77.7 (0.1)	81.4 (0.5)	89.8 (0.6)	95.9 (0.2)	89.0 (0.6)	82.8 (0.7)	78.7 (0.5)	79.3 (0.8)	79.4 (0.2)
**Dp-3-*trans*-*p­*cou-rut-5-glu**	78.0 (0.3)	83.0 (0.4)	94.3 (0.1)	98.3 (0.1)	97.4 (0.2)	93.1 (0.1)	75.3 (1.2)	78.1 (0.2)	79.3 (1.0)
**Pt-3-rut-5-glu**	74.6 (0.2)	82.3 (0.3)	94.9 (0.0)	98.2 (0.1)	98.4 (0.1)	97.3 (0.1)	77.2 (2.3)	72.1 (1.1)	70.3 (1.2)
**Pt-3-*cis*-*p­*cou-rut-5-glu**	81.1 (0.2)	83.7 (0.2)	92.0 (0.6)	97.3 (0.1)	98.1 (0.1)	96.9 (0.3)	83.5 (0.5)	83.3 (0.4)	83.7 (0.3)
**Pt-3-*trans*-*p­*cou-rut-5-glu**	76.1 (0.3)	82.5 (0.1)	93.5 (0.5)	97.9 (0.1)	98.3 (0.1)	92.3 (0.3)	73.4 (0.3)	73.5 (0.6)	70.4 (0.3)
Chroma (C*_ab_)									
**Dp-3-rut-5-glu**	58.3 (0.1)	41.3 (1.0)	12.2 (0.1)	3.1 (0.2)	2.0 (0.3)	2.3 (0.1)	9.0 (0.4)	22.3 (0.5)	16.2 (2.0)
**Dp-3-*cis*-*p­*cou-rut-5-glu**	50.2 (0.2)	42.4 (1.1)	21.8 (1.3)	4.0 (0.3)	10.1 (0.5)	17.0 (0.8)	23.8 (0.6)	24.2 (0.8)	22.8 (0.6)
**Dp-3-*trans*-*p­*cou-rut-5-glu**	48.4 (0.6)	38.2 (0.7)	11.7 (0.1)	2.0 (0.1)	1.9 (0.2)	6.4 (0.1)	28.2 (1.5)	28.4 (0.2)	35.1 (1.6)
**Pt-3-rut-5-glu**	58.6 (0.5)	42.5 (0.7)	10.6 (0.0)	2.4 (0.1)	1.5 (0.1)	1.9 (0.1)	22.2 (2.3)	37.5 (1.2)	39.0 (1.2)
**Pt-3-*cis*-*p*-cou-rut-5-glu**	44.5 (0.4)	38.4 (0.4)	16.0 (0.2)	3.3 (0.2)	1.2 (0.1)	1.3 (0.3)	19.2 (0.8)	27.9 (0.3)	29.9 (0.4)
**Pt-3-*trans*-*p­*cou-rut-5-glu**	52.0 (0.7)	38.8 (0.4)	12.4 (0.9)	2.5 (0.1)	1.0 (0)	9.5 (0.3)	33.0 (0.3)	34.2 (0.6)	36.9 (0.3)
**Hue** **angle (h_ab_)**									
**Dp-3-rut-5-glu**	0.3 ^c^ (0.1)	356.4 ^a^ (0.2)	359.0 ^a^ (0.3)	26.3 ^c^ (2.2)	55.5 ^c^ (2.6)	75.7 ^c^ (2.7)	272.3 ^b^ (1.1)	218.7 ^c^ (1.3)	198.4 ^c^ (5.3)
**Dp-3-*cis*-*p­*cou-rut-5-glu**	343.9 ^b^ (0.2)	344.0 ^c^ (0.2)	341.2 ^b^ (0.4)	327.6 ^b^ (2.8)	255.5 ^b^ (0.4)	247.5 ^b^ (0.3)	231.3 ^c^ (0.5)	223.2 ^b^ (0.3)	218.6 ^b^ (0.5)
**Dp-3-*trans*-*p­*cou-rut-5-glu**	344.5 ^a^ (0.1)	343.4 ^b^ (0.2)	341.7 ^b^ (0.1)	342.0 ^a^ (1.1)	293.9 ^a^ (3.1)	280.8 ^a^ (0.5)	283.6 ^a^ (1.1)	237.0 ^a^ (0.2)	236.8 ^a^ (1.7)
**Pt-3-rut-5-glu**	357.3 ^a^ (0.1)	354.2 ^a^ (0.1)	358.2 ^a^ 90.5)	34.5 ^c^ (2.0)	58.2 ^c^ (2.5)	33.7 ^c^ (1.5)	274.5 ^b^ (1.2)	231.5 ^b^ (0.7)	224.6 ^b^ (0.6)
**Pt-3-*cis*-*p*-cou-rut-5-glu**	341.2 ^c^ (0.1)	340.7 ^c^ (0.2)	336.6 ^c^ (3.2)	337.8 ^b^ (1.4)	325.0 ^b^ (3.6)	299.1 ^b^ (8.3)	246.3 ^c^ (0.2)	221.5 ^c^ (0.1)	215.0 ^c^ (0.9)
**Pt-3-*trans*-*p­*cou-rut-5-glu**	342.8 ^b^ (0.1)	342.2 ^b^ (0.1)	342.2 ^b^ (0.4)	352.6 ^a^ (1.0)	356.3 ^a^ (3.9)	322.3 ^c^ (1.1)	296.8 ^a^ (0.3)	250.1 ^a^ (0.4)	265.0 ^a^ (0.2)

**Table 3:** Half-lives (h) and rate constants (k) of delphinidin (Dp) and petunidin (Pt) derivatives in pH 1–9, *n* = 3. Different superscript letters indicate significant differences (*p* < 0.05) between derivatives of the same anthocyanidin in the same pH.

Anthocyanin	pH 1	pH 2	pH 3	pH 4	pH 5	pH 6	pH 7	pH 8	pH 9
**t_1/2_ (h)**									
**Dp-3-rut-5-glu**	675.0 ^b^ (9.3)	378.7 ^a^ (16.8)	102.7 ^a^ (6.2)	34.2 ^b^ (5.5)	* NM	* NM	1.7 ^c^ (0.0)	1.1 ^c^ (0.0)	0.7 ^c^ (0.0)
**Dp-3-*cis*-*p*-cou-rut-5-glu**	1077.2 ^a^ (21.6)	424.0 ^a^ (128.9)	52.9 ^b^ (0.6)	16.0^c^ (0.2)	13.08 ^b^ (0.2)	12.8 ^b^ (0.0)	12.6 ^a^ (0.1)	8.6 ^b^ (0.1)	7.5 ^b^ (0.4)
**Dp-3-*trans*-*p*-cou-rut-5-glu**	666.3 ^b^ (47.6)	457.5 ^a^ (26.2)	89.2 ^a^ (7.2)	53.9 ^a^ (1.6)	67.5 ^a^ (8.4)	18.3 ^a^ (7.7)	7.7 ^b^ (0.1)	13.2 ^a^ (0.7)	13.7 ^a^ (0.6)
**Pt-3-rut-5-glu**	183.9 ^b^ (26.0)	65.3 ^b^ (5.3)	28.5 ^a^ (1.9)	18.0(1.5)	19.5(2.5)	6.5 ^a^ (0.2)	1.7 ^b^ (0.0)	2.5 ^b^ (0.0)	1.1 ^c^ (0.0)
**Pt-3-*cis*-*p*-cou-rut-5-glu**	546.8 ^b^ (20.3)	137.4 ^a^ (19.8)	17.8 ^b^ (1.7)	* NM	* NM	1.9 ^b^ (0.1)	1.8^b^ (0.0)	3.3 ^b^ (0.1)	4.0 ^b^ (0.7)
**Pt-3-*trans*-*p*-cou-rut-5-glu**	2309.6 ^a^ (389.1)	109.7 ^a^ (11.8)	25.3 ^a^ (2.6)	* NM	* NM	7.1 ^a^ (0.8)	4.2 ^a^ (0.1)	7.3 ^a^ (0.6)	5.5 ^a^ (0.6)
**k**									
**Dp-3-rut-5-glu**	0.0010 (0.0000)	0.0018 (0.0001)	0.0068 (0.0004)	0.0206 (0.0032)	* NM	* NM	0.4004 (0.0034)	0.6110 (0.0078)	1.0377 (0.0583)
**Dp-3-*cis*-*p*-cou-rut-5-glu**	0.0006 (0.0000)	0.0017 (0.0005)	0.0131 (0.0001)	0.0433 (0.0006)	0.0532 (0.0001)	0.0540 (0.0001)	0.0552 (0.0005)	0.0808 (0.0005)	0.0929 (0.0049)
**Dp-3-*trans*-*p*-cou-rut-5-glu**	0.0010 (0.0001)	0.0015 (0.0001)	0.0078 (0.0007)	0.0129 (0.0004)	0.0104 (0.0012)	0.0379 (0.0015)	0.0900 (0.0009)	0.0527 (0.0028)	0.0507 (0.0020)
**Pt-3-rut-5-glu**	0.0038 (0.0001)	0.0107 (0.0001)	0.0244 (0.0016)	0.0386 (0.0032)	0.0359 (0.0049)	0.1073 (0.0029)	0.4019 (0.0064)	0.2780 (0.0033)	0.6163 (0.0071)
**Pt-3-*cis*-*p*-cou-rut-5-glu**	0.0013 (0.0000)	0.0051 (0.0007)	0.0391 (0.0040)	* NM	* NM	0.3745 (0.0124)	0.3832 (0.0091)	0.2127 (0.0054)	0.1751 (0.0329)
**Pt-3-*trans*-*p*-cou-rut-5-glu**	0.0003 (0.0001)	0.0064 (0.0001)	0.0277 (0.0030)	* NM	* NM	0.0988 (0.0118)	0.1664 (0.0029)	0.0956 (0.0070)	0.1273 (0.0122)

* NM: not measurable due to lack of absorbance at initial timepoint.

## References

[1] McCann D., Barrett A., Cooper A., Crumpler D., Dalen L., Grimshaw K., Kitchin E., Lok K., Porteous L., Prince E. (2007). Food additives and hyperactive behaviour in 3-year-old and 8/9-year-old children in the community: A randomised, double-blinded, placebo-controlled trial. Lancet.

[2] Potera C. (2010). The Artificial Food Dye Blues. Environ. Health Perspect..

[3] Dornblaser L., Jago D. (2013). Colors and Flavors: The move to more natural. Mintel.

[4] Sigurdson G., Tang P., Giusti M.M. (2017). Natural Colorants: Food Colorants from Natural Sources. Annu. Rev. Food Sci. Technol..

[5] Andersen Ø.M., Jordheim M., Wallace T.C., Giusti M.M. (2014). Basic Anthocyanin Chemistry and Dietary Sources. Anthocyanins in Health and Disease.

[6] Ananga A., Georgiev V., Ochieng J., Phills B., Tsolova V., Puljuha D., Sladonja B. (2013). Production of Anthocyanins in Grape Cell Cultures: A Potential Source of Raw Material for Pharmaceutical, Food, and Cosmetic Industries. The Mediterranean Genetic Code-Grapevine and Olive.

[7] Cesa S., Carradori S., Bellagamba G., Locatelli M., Casadei M.A., Masci A., Paolicelli P. (2017). Evaluation of processing effects on anthocyanin content and colour modifications of blueberry (*Vaccinium* spp.) extracts: Comparison between HPLC-DAD and CIELAB analyses. Food Chem..

[8] Rakic V., Skrt M., Miljkovic M., Kostic D., Sokolovic D., Poklar-Ulrih N. (2015). Effects of pH on the stability of cyanidin and cyanidin 3-*O*-β-glucopyranoside in aqueous solution. Hem. Ind..

[9] Zhao C.L., Chen Z.J., Bai X.S., Ding C., Long T.J., Wei F.G., Miao K.R. (2014). Structure-activity relationships of anthocyanidin glycosylation. Mol. Divers..

[10] Fossen T., Cabrita L., Andersen O.M. (1998). Colour and stability of pure anthocyanins influenced by pH including the alkaline region. Food Chem..

[11] Mazza G., Brouillard R. (1987). Recent developments in the stabilization of anthocyanins in food products. Food Chem..

[12] Torskangerpoll K., Andersen Ø.M. (2005). Colour stability of anthocyanins in aqueous solutions at various pH values. Food Chem..

[13] Stintzing F.C., Stintzing A.S., Carle R., Frei B., Wrolstad R.E. (2002). Color and antioxidant properties of cyanidin-based anthocyanin pigments. J. Agric. Food Chem..

[14] Giusti M.M., Wrolstad R.E. (2003). Acylated anthocyanins from edible sources and their applications in food systems. Biochem. Eng. J..

[15] Malien-Aubert C., Dangles O., Amiot M.J. (2001). Color stability of commercial anthocyanin-based extracts in relation to the phenolic composition. Protective effects by intra- and intermolecular copigmentation. J. Agric. Food Chem..

[16] Malcıoğlu O.B., Calzolari A., Gebauer R., Varsano D., Baroni S. (2011). Dielectric and Thermal Effects on the Optical Properties of Natural Dyes: A Case Study on Solvated Cyanin. J. Am. Chem. Soc..

[17] Ahmadiani N., Robbins R.J., Collins T.M., Giusti M.M. (2016). Molar absorptivity (ε) and spectral characteristics of cyanidin-based anthocyanins from red cabbage. Food Chem..

[18] George F., Figueiredo P., Toki K., Tatsuzawa F., Saito N., Brouillard R. (2001). Influence of *trans-cis* isomerisation of coumaric acid substituents on colour variance and stabilisation in anthocyanins. Phytochemistry.

[19] Yoshida K., Kondo T., Kameda K., Goto T. (1990). Structure of Anthocyanins Isolated from Purple Leaves of *Perilla ocimoides* L. var. crispa Benth and Their Isomerization by Irradiation of Light. Agric. Biol. Chem..

[20] Hosokawa K., Fukushi E., Kawabata J., Fujii C., Ito T., Yamamura S. (1997). Seven acylated anthocyanins in blue flowers of Hyacinthus orientalis. Phytochemistry.

[21] Ichiyanagi T., Kashiwada Y., Shida Y., Ikeshiro Y., Kaneyuki T., Konishi T. (2005). Nasunin from Eggplant Consists of *Cis−Trans* Isomers of Delphinidin 3-[4-(*p*-Coumaroyl)-l-rhamnosyl(1→6)glucopyranoside]-5-glucopyranoside. J. Agric. Food Chem..

[22] Zheng J., Ding C., Wang L., Li G., Shi J., Li H., Wang H., Suo Y. (2011). Anthocyanins composition and antioxidant activity of wild *Lycium ruthenicum* Murr. from Qinghai-Tibet Plateau. Food Chem..

[23] Inami O., Tamura I., Kikuzaki H., Nakatami N. (1996). Stability of anthocyanins of Sambucus canadensis and Sambucus nigra. J. Agric. Food Chem..

[24] Jin H., Liu Y., Guo Z., Yang F., Wang J., Li X., Peng X., Liang X. (2015). High-performance liquid chromatography separation of *cis*–*trans* anthocyanin isomers from wild *Lycium ruthenicum* Murr. employing a mixed-mode reversed-phase/strong anion-exchange stationary phase. J. Agric. Food Chem..

[25] Harborne J.B. (1967). Comparative Biochemistry of the Flavonoids.

[26] Brouillard R., Delaporte B. (1977). Chemistry of Anthocyanin Pigments. 2. * Kinetic and Thermodynamic Study of Proton Transfer, Hydration, and Tautomeric Reactions of Malvidin 3-Glucoside. J. Am. Chem. Soc..

[27] Sigurdson G.T., Robbins R.J., Collins T.M., Giusti M.M. (2017). Spectral and colorimetric characteristics of metal chelates of acylated cyanidin derivatives. Food Chem..

[28] Yoshida K., Mori M., Kondo T. (2009). Blue flower color development by anthocyanins: from chemical structure to cell physiology. Nat. Prod. Rep..

[29] Sigurdson G.T., Giusti M.M. (2014). Bathochromic and Hyperchromic Effects of Aluminum Salt Complexation by Anthocyanins from Edible Sources for Blue Color Development. J. Agric. Food Chem..

[30] Asenstorfer R.E., Iland P.G., Tate M.E., Jones G.P. (2003). Charge equilibria and pKa of malvidin-3-glucoside by electrophoresis. Anal. Biochem..

[31] León-Carmona J.R., Galano A., Alvarez-Idaboy J.R. (2016). Deprotonation routes of anthocyanidins in aqueous solution, pKa values, and speciation under physiological conditions. RSC Adv..

[32] Rodríguez-Saona L.E., Wrolstad R.E., Wrolstad R.E., Acree T.E., An H., Decker E.A., Penner M.H., Reid D.S., Schwartz S.J., Shoemaker C.F., Sporns P. (2001). Extraction, Isolation, and Purifification of Anthocyanins. Current Protocols in Food Analytical Chemistry.

[33] Giusti M.M., Wrolstad R.E., Wrolstad R.E., Acree T.E., An H., Decker E.A., Penner M.H., Reid D.S., Schwartz S.J., Shoemaker C.F., Sporns P. (2001). Separation and Characterization of Anthocyanins by HPLC. Current Protocols in Food Analytical Chemistry.

[34] Azuma K., Ohyama A., Ippoushi K., Ichiyanagi T., Takeuchi A., Saito T., Fukuoka H. (2008). Structures and antioxidant activity of anthocyanins in many accessions of eggplant and its related species. J. Agric. Food Chem..

[35] Sadilova E., Stintzing F.C., Carle R. (2006). Anthocyanins, colour and antioxidant properties of eggplant (*Solanum melongena* L.) and violet pepper (*Capsicum annuum* L.) peel extracts. Z. Naturforsch. Sect. C J. Biosci..

[36] Giusti M.M., Wrolstad R., Wrolstad R.E., Acree T.E., An H., Decker E.A., Penner M.H., Reid D.S., Schwartz S.J., Shoemaker C.F., Sporns P. (2001). Characterization and measurement of anthocyanins by UV- Visible spectroscopy. Current Protocols in Food Analytical Chemistry.

[37] Farr J.E., Giusti M.M. ColorbySpectra-Academic License. https://buckeyevault.com/products/colorbyspectra.

